# Lentivirus-mediated short hairpin RNA interference targeting TNF-alpha in macrophages inhibits particle-induced inflammation and osteolysis in vitro and in vivo

**DOI:** 10.1186/s12891-016-1290-6

**Published:** 2016-10-18

**Authors:** Chu-qiang Qin, Dong-sheng Huang, Chi Zhang, Bin Song, Jian-bin Huang, Yue Ding

**Affiliations:** Department of Orthopaedic Surgery, The Memorial Hospital of Sun Yat-Sen University, 107 Yanjiangxi Road, 510120 Guangzhou, China

**Keywords:** Hip arthroplasty, Periprosthetic osteolysis, Lentivirus, RNA interference, Wear particle

## Abstract

**Background:**

Aseptic loosening is a significant impediment to joint implant longevity. Prosthetic wear particles are postulated to play a central role in the onset and progression of periprosthetic osteolysis, leading to aseptic loosening of the prosthesis.

**Methods:**

We investigated the inhibitory effects of a lentivirus-mediated short hairpin RNA that targets the TNF-alpha gene on the particle-induced inflammatory and osteolytic changes via macrophages both in vitro and in vivo. An siRNA sequence targeting the mouse TNF-alpha gene from four candidates, transcribed in vitro, was screened and identified. A lentivirus vector expressing short hairpin RNA (shRNA) was then constructed in order to facilitate efficient expression of TNF-alpha-siRNA. Lentivirus-mediated shRNA was transduced into cells of the mouse macrophage line RAW 264.7. Ceramic and titanium particles were introduced 24 h after lentivirus transduction to stimulate cells. TNF-alpha expression, represented by both mRNA and protein levels, was quantified with real-time PCR and ELISA at all time intervals. Lentivirus-mediated shRNA suspension was locally administered into the murine calvarial model, followed by local injection of particles. A multi-slice spiral CT scan was used to evaluate the osteolysis of the calvaria by detecting the width of the cranial sutures.

**Results:**

Macrophages developed pseudopods when co-cultured with particles. Lentivirus-mediated shRNA was shown to effectively inhibit the expression of TNF-alpha at both the mRNA and protein levels in RAW 264.7. The multi-slice spiral CT scan showed that the lentivirus-mediated shRNA significantly suppressed osteolysis of mouse calvaria.

**Conclusions:**

Our investigation highlighted the results that lentivirus-mediated shRNA targeting the TNF-alpha gene successfully inhibited particle-induced inflammatory and osteolytic changes both in vitro and in vivo. Therefore, lentivirus-mediated gene therapy may provide a novel therapeutic approach to aseptic joint loosening.

## Background

Total joint replacement is a common procedure and has been proven to be highly successful for the treatment of advanced arthropathy. Internationally, nearly 1.5 million total arthroplasties are performed every year [1]. Although considerable efforts have been made to improve the efficacy and quality of total joint replacement, there are still numerous challenges facing the long-term success of arthroplastic surgery. Aseptic prosthetic loosening constitutes one of the most common causes of arthroplasty failure [1]. Additionally, there are, as yet, no approved conservative management methods to prevent or inhibit periprosthetic osteolysis. With increasing life expectancy and arthroplasties becoming more common in younger and more active patients, osteolytic aseptic loosening has risen to become the principle cause for revision surgery. Understanding the exact mechanism of aseptic loosening and developing solutions to contain the loosening process are thus a critical and urgent task. Recent attempts have been directed at understanding and changing osteolysis through intervention at the molecular level.

Wearing a prosthesis is believed to play a leading role in the onset and progression of periprosthetic osteolysis. The particles resulting from internal wear induce osteolysis, as has been evidenced by in vitro studies, as well as in various animal models [2]. Titanium-alloy components have been broadly used in total joint replacement [3]. Particulate titanium debris is one of the most frequently seen metal debris types in tissues at a failed joint prosthesis. As an emergent material, ceramic has also been widely used in total joint replacement [4].

Aseptic loosening is believed to be initiated by the interactions between periprosthetic cells and wear particles. It has also been highlighted that macrophages make up 60–80 % of the entire cellular population around loosening periprosthetic tissues [5]. Inflammatory cytokines contribute to the progression of wear particle-induced inflammatory osteolysis, including TNF-α, IL-1β, IL-6, and IL-10 [6]. These inflammatory cytokines further become direct or indirect stimulants to osteoclast attachment, differentiation, activation, and maturation, resulting in bone resorption around implants and possible implant failure [7]. TNF-alpha has been shown to play an important role in osteoclastogenesis and osteolysis [8]. This is supported by the fact that etanercept, a soluble inhibitor of TNF-a, reduces wear debris particle-induced bone resorption and osteoclast formation to a background level in both normal mice and those transferred with human TNF-alpha (hTNF-alpha), a phenomenon which was also observed in a bone wafer pit assay [9]. These studies all strongly suggest that this could be a promising way to inhibit osteoclastogenesis and osteolysis induced by wear debris particles by limiting the TNF-alpha-releasing macrophages. Antagonist, antibody, and bisphosphonate therapies have the disadvantages of high dose and frequency of administration. In particular, several studies have shown that the nitrogen-containing bisphosphonates, such as alendronate and zoledronate, have been shown to inhibit the osteolysis resulting from wear debris, as well as to increase bone mineral density around implants [10], but their side-effects, such as fever and throat and stomach ulcers, as well as their low bioavailability, constitute challenges for systemic treatment [11]. These limitations have increased the demand for a better TNF-alpha inhibitor with improved potency, sustainability, and safety.

As a post-transcriptional gene silencing mechanism, RNA interference (RNAi) has demonstrated great promise for the study of human gene function, signal transduction research, and gene therapy [12]. Recently, RNAi–based approaches involving small interfering RNA (siRNA) and short hairpin RNA (shRNA) have been widely utilized to silence the expression of many target genes. However, there are major barriers to the possible therapeutic use of RNAi. The most apparent challenges for the use of siRNA are inefficient transfection in primary cells and the transient knockdown effect. In contrast, shRNA provides a more sustained inhibitory effect than chemically synthesized siRNA. Nevertheless, some disadvantages remain, including the lack of sustained shRNA expression and an effective and nontoxic in vivo delivery system. To overcome these limitations, several attempts have been made to this end, such as the selection of conserved viral sequences to be RNAi targets and the development of viral delivery systems that express siRNA or shRNA [13]. Among these delivery systems, the lentivirus is an attractive option, because it is a well-established vehicle for in vivo gene transfer and is capable of stably transducing cells in both dividing and quiescent phases [14]. In addition to its ability to stably integrate into the host’s genome, the remarkable packaging capacity of lentiviral vectors also renders them to be effective gene transfer tools [15]. Additionally, lentiviral vectors show minimal immunogenicity [16]. Lentivirus vectors encoding anti-sense targeting sequences have been shown to be free from obvious side effects in clinical trials [17]. Therefore, the lentiviral vector is a suitable delivery method for RNAi technology in order to facilitate the development of therapeutic strategies.

In the present study, we aimed to find an efficient system by fabricating a recombined lentivirus vector encoding shRNA targeted against TNF-alpha and examining the TNF-alpha knockdown efficiency in mouse macrophages, as well as the in vivo effect on reducing the osteolysis and inflammation seen in mice with experimental particle-induced osteolysis.

## Methods

### Particle preparation

Commercially pure titanium particles of 4.507 g/cm^3^ were purchased from Alfa Aesar (Ward Hill, MA, USA), and alumina ceramic particles of 3.53 g/cm^3^ from Ceram Tec (Plochingen, Germany). All particles were disseminated in pure water and filtered through Millipore filter membranes (Billerica, MA, USA) of different diameters (0.2 μm and 1.2 μm) to select particles with a target size between 0.2 μm and 1.2 μm. An image analysis system (NIKON, Japan) was used to measure the exact size of the particles, and the results were 0.82 ± 0.12 μm and 0.84 ± 0.14 μm for the filtrated titanium and ceramic particles, respectively. All particles were rinsed with 70 % ethanol for 24 h at room temperature and then dried in an oven before being sterilized with ethylene oxide. Based on particle weight and density, particles incubated in phosphate buffered saline (PBS) were adjusted to 4 × 10^8^ μm^3^/ml in concentration. The level of endotoxin in particle solutions was detected using the Limulus Amebocyte Lysate Assay (QCL-1000; Bio Whittaker, Walkersville, Maryland, USA), and the result was below the detection level of 0.01 EU/ml.

### Construction of lentiviral vector expressing TNF-alpha-specific shRNA

Four siRNA sequences against the mouse TNF-alpha gene (Gene Bank Accession No NM_004517.2) were designed using online siRNA software. Preliminary experiments indicated that the best performing TNF-alpha siRNA sequence with the highest inhibitory efficiency was 5’-GCAAACAGAGCATGGTCAA-3’. Meanwhile, a missense siRNA (MS siRNA): 5’-TAATCGTCGT AGACGGTTG-3’ was used as the control. Based on the siRNA sequences described above, an shRNA cassette was designed with 19 nucleotides (nt) of the target sequence, a loop sequence (TTCAAGAGA), the reverse complement of the 19-nt target sequence, and then a stop codon for the U6 promoter, as well as digestion sites for HpaI and XhoI. Following the design described above, the shRNA cassettes were synthesized along with their complementary strands. The shRNAs were inserted into the pFU-GW-iRNA lentivirus vector, which contained a CMV-driven EGFP reporter gene and a U6 promoter upstream from the restriction sites (HpaI and XhoI). All of the constructs were ascertained by sequence analysis. The vector was designed to co-express green fluorescent protein (GFP) cloned from a copepod. Bearing the shRNA sequences and pPACK Packaging Plasmid Mix (System Biosciences), the pFU-GW-iRNA lentivirus vector was co-transfected into 293TN cells with Lipofectamine 2000 (Invitrogen). After 48 h, viral supernatants were harvested and passed through 0.45 μm filters. The target lentivirus and the negative control lentivirus expressing TNF-alpha-siRNA and non-targeting sequence were denoted as TNF-alpha-RNAi-LV and NC-GFP-LV, respectively. Expression levels of GFP were studied by FACS analysis and PCR. In order to calculate the virus titers, the number of cells was multiplied by both the percentage of GFP-positive cells and the dilution factor, resulting in the formation of the titer units (TU)/ml. The concentration was 5 × 10^8^ TU (titer unit)/ml.

### Cell culture and lentivirus transduction

RAW264.7, the murine macrophage cell line, was obtained from American Type Culture Collection (ATCC Number: TIB-71). The cells were seeded and cultured in 96-well tissue culture plates (Costar, Cambridge, MA, USA) in DMEM medium with 10 % fetal bovine serum (FBS) (Hyclone). Cells were incubated overnight in a humidified incubator at 37 °C with 5 % CO_2_ to improve the adherence of cells to the plates. Trypan blue dye exclusion was used for cell quantitation and the viability assays, with viability exceeding 98 % for all trials.

For lentivirus transduction, RAW 264.7 macrophages were infected with either TNF-alpha-RNAi-LV or NC-GFP-LV in serum-free growth medium with 5 mg/ml polybrene at multiplicities of infection (MOI) of 150, which was optimized from the toxicity curve before transduction. After 8 h’ of culturing, the medium was substituted by fresh DMEM complete medium, in order to remove debris and inactive lentiviruses before the cells were continuously incubated for another 72 h. Three days after transfection, the transfection efficiency was examined by fluorescent microscopy.

### Particle stimulation and molecular analysis of cytokines expression

After lentivirus transduction, cells seeded in 6-well plates were digested. The particles were added to all of the experimental groups, except for the control group. They were then developed into suspensions at a final concentration of 100:1 (particle volume μm^3^: cell number) and supplemented to cultures, whereupon the culture plates were gently agitated for 10 min. Experimental grouping was made up of seven groups, as follows:

- Control Group: free of any interference measure or particle stimulation
- TI Group: free of any interference measure, but stimulated with titanium particles
- TI + TNF-LV Group: transfected with TNF-alpha-RNAi-LV and stimulated with titanium particles
- TI + N.C-LV Group: transfected with NC-GFP-LV and stimulated with titanium particles
- CE Group: free of any interference measure, but stimulated with ceramic particles
- CE + TNF-LV Group: transfected with TNF-alpha-RNAi-LV and stimulated with ceramic particles
- CE + N.C-LV Group: transfected with NC-GFP-LV and stimulated with ceramic particles

Experiments were performed three times in each group. After particle stimulation, the mRNA and supernatant were collected (0.5 h, 3 h, and 6 h for mRNA, and 6 h, 12 h, 24 h, and 48 h for supernatant), in order to determine interference efficiency by real-time PCR and ELISA.

An RNA-to-cDNA reverse transcription was performed with Primescript RT reagent (TaKaRa, Kyoto, Japan). The mRNA levels were determined using real-time PCR on the Roche Light Cycler 480 System (Roche, Mannheim, Germany). With Primer 5.0, the following gene-specific primers were designed (5’ to 3’): GAPDH, TGTGTCCGTCGTGGATCTGA (forward) and TTGCTGTTGAAGTCGCAGGAG (reverse), as well as TNF-alpha, AGCCCCCAGTCTGTATCCTT (forward) and CTCCCTTTGCAGAACTCAGG (reverse). The product was detected with the SYBR green dye, SYBR Premix Ex Taq (TaKaRa, Kyoto, Japan). The results of GAPDH amplification efficiency and TNF-alpha primers were 1.976 and 2.022, respectively. The comparative CT (threshold cycle) method with arithmetic formulas was used to determine the relative level of gene expression for both the target and house-keeping genes. Since the negative controls are referred to as the calibration factor, all of the other quantities are expressed as an *n* fold difference relative to the negative control.

The protein production of TNF-alpha in the supernatants was assayed with an immunoassay ELISA kit (Invitrogen, Carlsbad, CA, USA) as per the manufacturer’s instructions.

### Murine calvarial model

Male C57BL/6 mice, aged 12 weeks, were purchased from the Center of Animal Experiments of Sun Yat-sen University (Guangzhou, China). All animal handling and experiment procedures were performed in compliance with the guidelines of the National Institutes of Health for animal care, and all animal experiments were approved by The Memorial Hospital of Sun Yat-Sen University.

In this study, 49 mice were randomized into seven groups, with seven mice in each group:

- Control: PBS was administered locally into the cranial sutures without lentivirus infection or particle stimulation
- TI: stimulated with titanium particles, free from interference measures
- TI + TNF-LV: stimulated with titanium particles and transfected with TNF-alpha-RNAi-LV.
- TI + N.C-LV: stimulated with titanium particles and transfected with NC-GFP-LV
- CE: stimulated with ceramic particles, free from interference measures
- CE + TNF-LV: stimulated with ceramic particles and transfected with TNF-alpha-RNAi-LV
- CE + N.C-LV: stimulated with ceramic particles and transfected with N.C control-lentivirus mediated shRNA

Locally injecting wear particles in mice can establish a model of particle-induced osteolysis [18]. A 1*1 cm median incision was made on the surface of murine calvaria. Periosteum stripping was performed to fully expose the cranial sutures. A piece of 0.7*0.7 cm medical gelfoam was placed in order to medially cling to the surface of the calvaria. A 500 μl particle suspension (4 × 108 μm3/ml) was slowly injected into the intervals between the cranial sutures and the gelfoam to provoke an osteolytic response. The skin was then sutured. After 24 h, 0.5 ml of culture medium containing 2 × 10^7^ TU of TNF-alpha-RNAi-LV, NC-GFP-LV, or PBS alone was slowly injected into the intervals between the cranial sutures and the gelfoam, using a 1 ml injector. Since preliminary experiments have shown that particles injected locally on the surface of murine calvaria induced significant osteolysis by the 14th day, the mice in our study were sacrificed with carbon dioxide asphyxiation for CT scan analysis 14 days after transfection. All experiments involving the construction and in vivo evaluation of the lentiviral vectors were completed under bio-safety level II containment.

### CT scan analysis

**Fig. 1:**
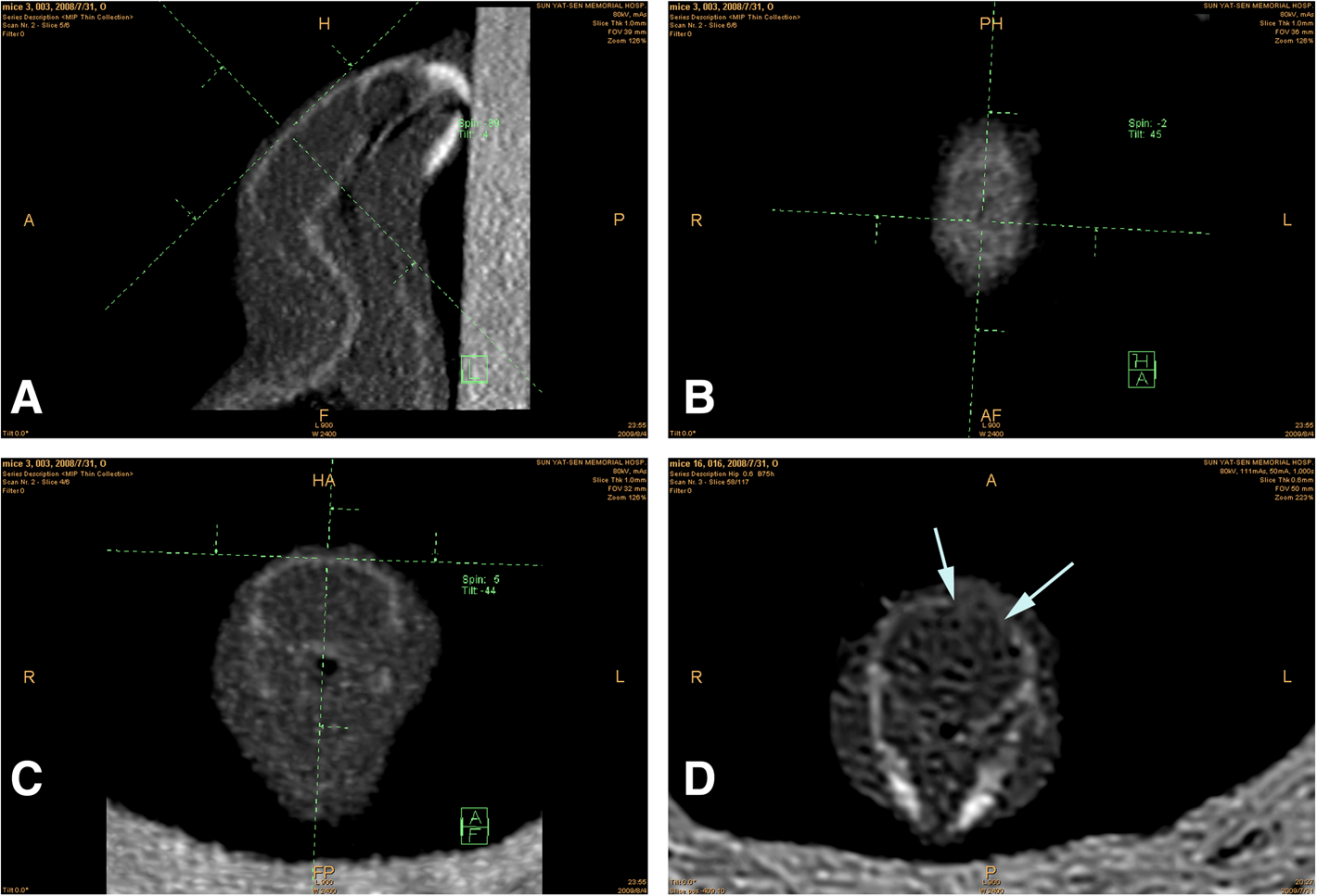
Measurement of cranial suture on orthogonal MPR images. The thin-slice images were reformatted with the MPR technique. The orthogonal sagittal (**a**), axial (**b**), and coronal planes (**c**) of the murine head were obtained. On the orthogonal coronal image (**d**), the largest cranial suture width (between the two *arrows*) was determined and measured

The osteolysis of the calvaria was evaluated based on the width of the cranial sutures after 2 weeks using multi-slice spiral CT scans (Fig. 1). The cranial CT scans were performed on a 64-slice spiral CT (Sensation 64, Siemens Medical Systems, Germany). The parameters of each scan were set at an axial collimation of 12 × 0.6 mm, a pitch of 0.8, a tubular voltage of 80 KV, and a tubular current of 120 mAs. The raw data obtained were reconstructed into contiguous 0.6-mm thick slices at 0.3 mm increments, with a field of view of 30 mm × 30 mm and a matrix of 512 × 512, using a B40 soft tissue, as well as a U75 bone algorithm. These two sets of thin-slice images were respectively post-processed by volume rendering (VR) and multi-planar reformation (MPR) to evaluate the osteolysis of calvaria and to perform related measurements. The detailed morphology of cranial sutures was observed by three-dimensionally displayed cranial sutures on a VR image, using appropriate threshold values. The MPR planes were first adjusted to achieve orthogonal three-dimensional cranial MPR images. Then the largest cranial suture width was measured on the obtained orthogonal coronal images.

### Statistical analysis

Results are shown as mean ± SD. ANOVA was performed to analyze differences between groups. Significance was set at a *p* value of < 0.05. All statistical analyses were performed using SPSS (version 16.0; SPSS, Chicago, IL, USA).

## Results

### Macrophages co-cultured with wear particles

**Fig. 2:**
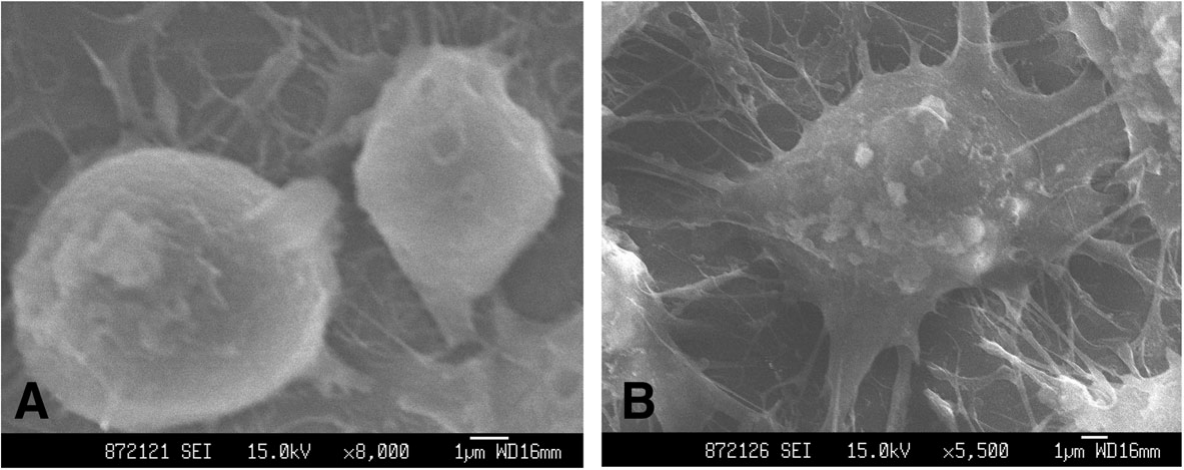
Macrophage and particles under scanning electron microscopy. **a** Non-stimulated macrophage alone (8000×), **b** Macrophages stimulated with titanium particles (0.82 ± 0.12 μm) (5500×)

A scanning electron microscope (SM-6330 F-Field Emission, JEOL, Japan) was used to observe the macrophages after particle stimulation, which showed that the macrophages in a stimulation-free environment basically maintained smooth and non-distorted contours (Fig. 2a). When co-cultured with particles, however, macrophages developed pseudopods (Fig. 2b).

### Transfection efficiency of lentivirus-mediated shRNA in RAW264.7macrophages

**Fig. 3:**
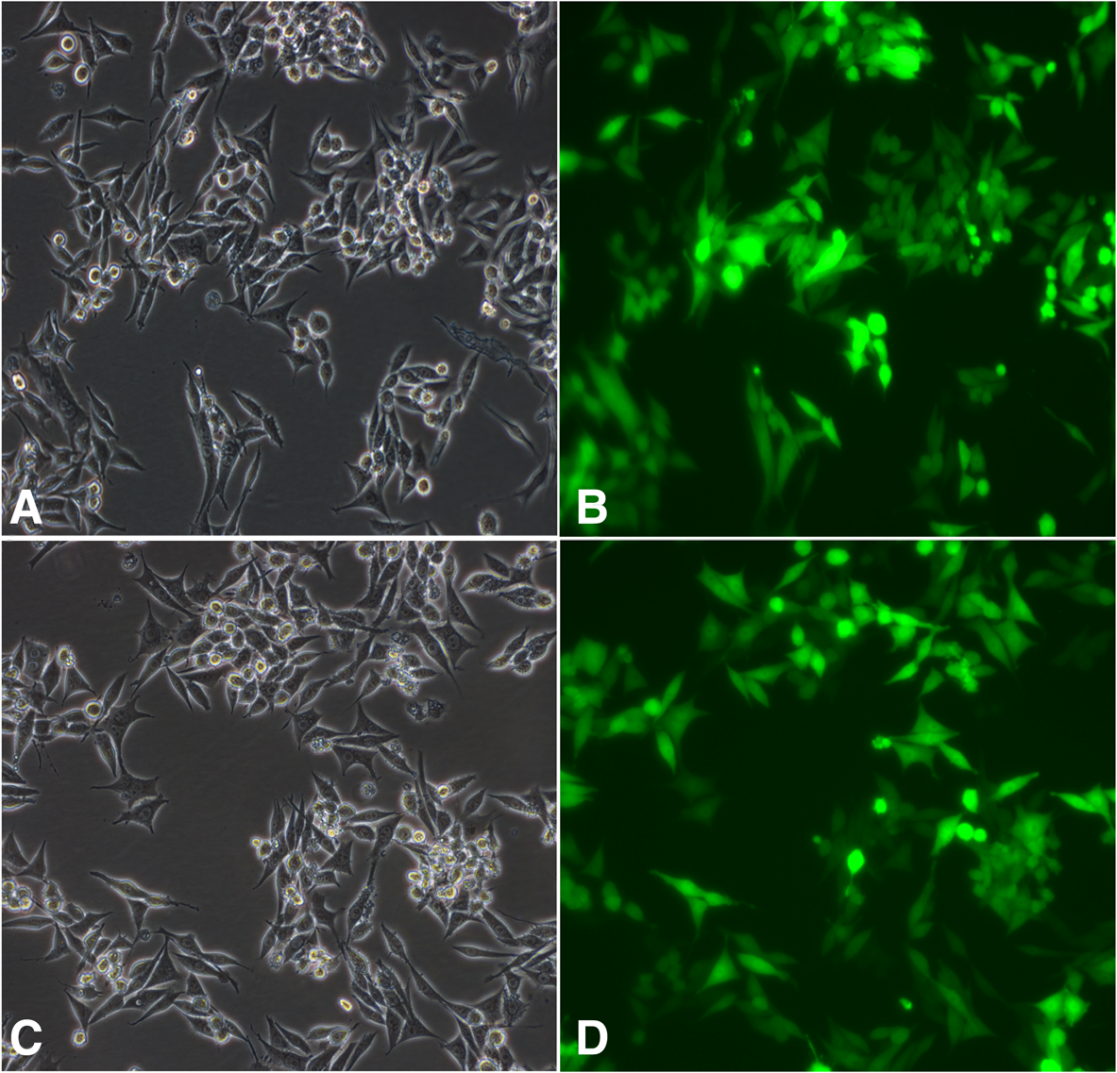
Transfection efficiency of lentivirus-mediated shRNA interference determined by fluorescence microscopy analysis. **a** and **c** transfected macrophage cells under light microscopy of shRNA-LV and NC-LV group (20×); **b** and **d** transfected macrophage cells under fluorescence microscopy of the same visual field of shRNA-LV and NC-LV group (20×)

The efficiency of lentivirus-mediated shRNA transfection into RAW264.7 macrophages was determined by GFP fluorescence. The numbers of cells and fluorescent spots in the same visual field were counted with light and fluorescence microscopes, respectively. The transfection efficiency of lentivirus-mediated shRNA via cationic liposomes was 85.2 ± 3.5 % (Fig. 3).

### Significant down-regulation of TNF-alpha mRNA in RAW264.7 macrophages by lentivirus-mediated shRNA

**Fig. 4:**
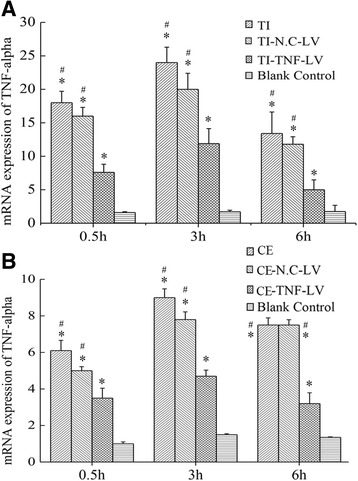
Multiple comparison tests of TNF-alpha mRNA levels at different time points. mRNA levels of TNF-alpha in transfected macrophage cells at 3 h after particle stimulation. *p < 0.05 When compared to the control group; *p < 0.05 when compared to the Particle + TNF-LV group. **a** Titanium particles treatment; **b** Ceramic particles treatment

The results of multiple comparisons among different time points demonstrated that the TNF-alpha mRNA was significantly reduced at 0.5 h, 3 h, and 6 h after transfection, having reached peak reduction at the 3 h time point (Fig. 4a, b). The down-regulation of TNF-alpha mRNA in the Particle + TNF-LV group was significant when compared with the Particle and Particle + N.C-LV groups (*p* < 0.05) at any time point, and was insignificant between the Particle and Particle + N.C-LV groups (*p* > 0.05) (Fig. 4a, b). The inhibition ratios [(mRNA levels of the Particle group minus those of the Particle + TNF-LV group)/Particle group] are available in Table 1. Results also revealed that TNF-alpha expression in TNF-LV-transfected macrophages was suppressed by 53.22 and 46.52 % for titanium and ceramic particles, respectively, 3 h after the introduction of particles.

**Table 1:** Inhibition ratios of mRNA levels and protein levels

Particles	mRNA levels mean ± SD				protein levels mean ± SD (pg/ml)			
	Particle	Particle + TNF-LV	Particle + N.C-LV	IR^a^	Particle	Particle + TNF-LV	Particle + N.C-LV	IR^a^
TI	24.06 ± 5.06	11.26 ± 3.48	1.50 ± 0.57	53.22 %	780.12 ± 36.36	490.03 ± 8.80	730.06 ± 45.88	37.18 %
CE	9.01 ± 1.49	4.82 ± 0.22	1.50 ± 0.57	46.52 %	572.11 ± 38.41	329.73 ± 17.22	527.11 ± 44.68	42.37 %

^a^IR (Inhibition ratio) = (mRNA or protein levels of Group Particle minus Group Particle + TNF-LV)/Group Particle. There were no significant differences in RNAi inhibitory effects among titanium and ceramic particle stimulation groups in both mRNA levels and protein levels (*P* > 0.05)

### Significant down-regulation of TNF-alpha protein in RAW264.7 macrophages by lentivirus-mediated shRNA

**Fig. 5:**
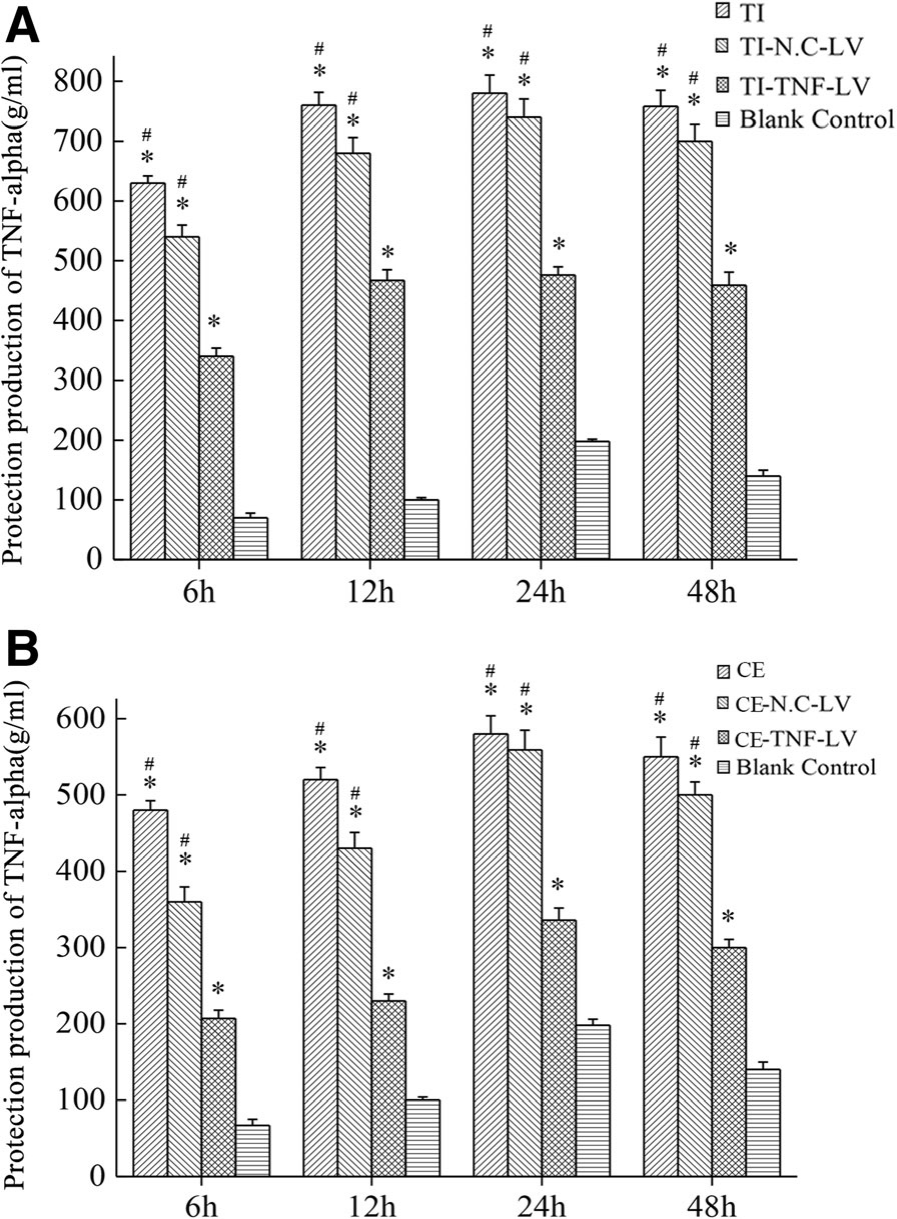
Multiple comparison tests of TNF-alpha protein levels for different time points. Protein levels of TNF-alpha in transfected macrophage cells at 24 h after particle stimulation. *p < 0.05 When compared to the control group; *p < 0.05 when compared to the Particle + TNF-LV group. **a** Titanium particles treatment; **b** Ceramic particles treatment

ELISA analysis of the cell supernatants demonstrated TNF-alpha gene silencing (Fig. 5a, b). As shown, TNF-alpha levels increased remarkably in the Particle group compared with the control, 6 h after stimulation began, and reached the peak after 24 h. We assayed protein levels among the experiment groups at the 4 time points for the RNAi inhibitory effects. The down-regulation of TNF-alpha protein in the Particle + TNF-LV group was significantly lower compared with the Particle and Particle + N.C-LV groups (*p* < 0.05), and was insignificant between the Particle and Particle + N.C-LV groups (*p* > 0.05) (Fig. 5a, b). The inhibition ratios for the Particle + TNF-LV group are displayed in Table 1. In addition, TNF-alpha protein levels in TNF-LV-transfected macrophages were suppressed by 37.18 and 42.37 % with titanium and ceramic particles, respectively. Finally, when the TI + TNF-LV and CE + TNF-LV groups were compared at the same protein levels for RNAi inhibitory effects, the results concluded no significant difference in RNA interference effects (*p* > 0.05).

### Inhibited particle-induced osteolysis in vivo, due to lentivirus-mediated TNF-alpha shRNA

**Fig. 6:**
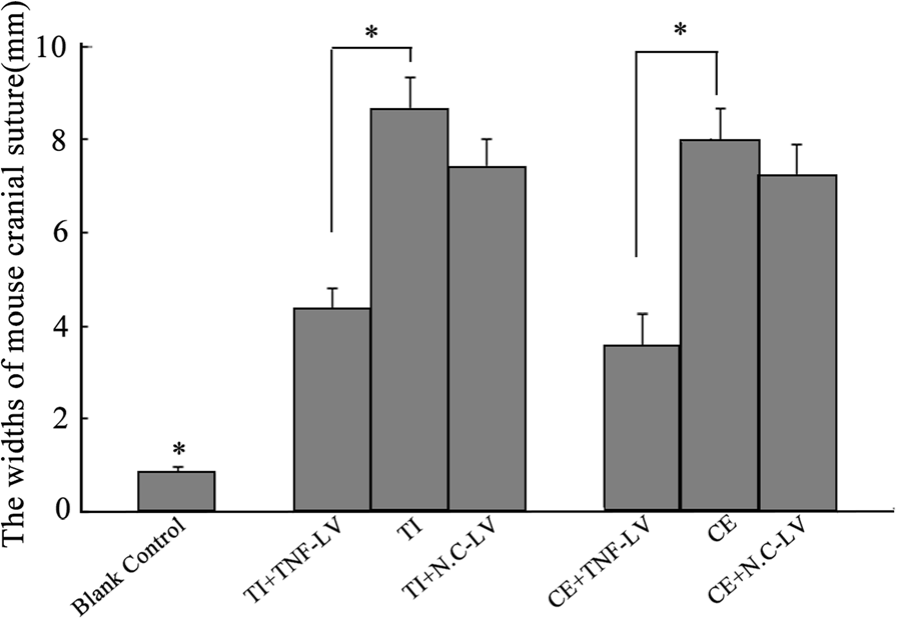
The width of the murine cranial suture was measured on the obtained orthogonal coronal images by 64-slice spiral CT. Significant reduction of cranial suture width was revealed in the Particle + TNF-LV group compared with the Particle and Particle + N.C-LV groups (*P* < 0.01). There was no difference between the Particle and Particle + N.C-LV groups in this parameter (*P* > 0.05)

The width of the cranial suture at the widest point was measured in order to assess the particle-induced osteolysis in the murine calvarial model. Significant narrowing of cranial suture width was revealed in the Particle + TNF-LV group compared with the Particle and Particle + N.C-LV groups (*p* < 0.01) (Fig. 6), suggesting that lentivirus-mediated shRNA interference reduced the osteolysis by approximately 40 % in the Particle + TNF-LV group. The width difference was negligible between the Particle and Particle + N.C-LV groups (*p* > 0.05).

## Discussion

Inflammation and the consequent osteolysis due to the presence of wear particles are both believed to be involved in the pathogenesis of aseptic loosening [19], which is, unfortunately, amenable by no other means than surgical revision. Efforts to prevent osteolysis have focused on improvements in implant design, biomaterials, or surgical techniques [20]. These approaches are insufficient, however, in regards to eliminating aseptic loosening, due to their inability to completely block particle generation from the bearing surfaces. Recently, a new approach was identified, starting with understanding and alleviating inflammatory osteolysis through pharmacological intervention [21]. Nevertheless, the delivery of an adequate level of cell specific therapy to the site of periprosthetic inflammation without causing systemic side effects is a formidable challenge. Researchers have been motivated by the current unsatisfactory methods for prevention and treatment of aseptic loosening to experiment and gain further insights from progress that has already been made in the study of the mechanisms of inflammation. Most previous studies have focused on methods for reducing inflammation caused by wear particles, while a few have investigated whether down-regulated inflammation could inhibit osteolysis.

Macrophages play a pivotal role in these processes [22]. Macrophages phagocytose implant-derived wear particles, leading to cytokine secretion, foreign-body-associated granulomatous and inflammatory reactions, osteoclastogenesis, activation, and subsequent osteoclastic bone resorption [23]. In the presence of wear debris particles, macrophages produce many cytokines, some of which stimulate the differentiation of osteoclast precursors, while others activate the mature osteoclasts [24]. The pro-inflammatory TNF-alpha is the dominant cytokine found around prostheses, and is known as a potent generator of bone resorption related to aseptic loosening [25]. It fuels osteoclast differentiation by up-regulating receptor activator nuclear factor κB ligand (RANKL) in stromal cells, as well as the macrophage colony stimulating factor (M-CSF) of macrophages, and enhancing the sensitivity of osteoclast precursors to RANKL [26]. In addition, TNF-alpha has been demonstrated to act directly on certain genes and their products, a feature crucial for osteoclast formation, differentiation, and activation [27]. The feasibility of inhibiting inflammation by blocking TNF-alpha has been evidenced in several therapies. For example, anti-TNF therapeutics have already been proven to be effective in fighting rheumatoid arthritis, a chronic inflammatory bone disease characterized by bone damage and elevated osteoclastic bone resorption around the affected joints [28]. There are, however, significant disadvantages to such therapy [29].

RNAi is a useful target for the analysis of gene function and a candidate for potential therapeutic strategies in various conditions [30]. The delivery of SiRNA into cells represents the bottleneck for RNAi therapy. Two mechanisms prevail in RNAi, namely the direct delivery of chemically synthesized siRNA into cells or the continuous production of siRNAs within cells via transcription. Chemically synthesized siRNA can be introduced into cells via conventional means, such as liposomes, polyethylenimine, or electroporation. However, the short-lived knockdown effect of these techniques put them at a disadvantage. More recently, therefore, the attention of many researchers has been drawn to expression vectors that enable mammalian cells to synthesize their own siRNA. A number of viral systems are being developed for ex vivo and in vivo gene transfer, among which are retroviruses, adenoviruses, herpes-simplex viruses, and adeno-associated viruses [31]. In the present study, a lentivirus vector was selected as the shRNA delivery vehicle due to its ability to infect both dividing and non-dividing cells with high efficiency, and to produce sustained gene expression by integrating into the host genome [32]. This methodology makes it possible for cells to continuously synthesize siRNA, an important step for the long-term silencing of a target gene [33]. The current study has proven the feasibility and efficacy of LV-mediated siRNA in hindering particle-induced inflammation. LV-shRNA was successfully constructed and proven safe for macrophages, and the LV-mediated TNF-alpha siRNA was shown to inhibit TNF-alpha expression with high efficiency, at both mRNA and protein levels. Furthermore, LV-shRNA brought about a significant hypoexpression of TNF-alpha, thereby alleviating osteolysis in murine calvaria. Our study justifies the use of lentivirus in aseptic loosening gene therapy studies. As indicated by subsequent experiments, this lentiviral transgenic system can be effectively transfected into RAW264.7 macrophages, with 85 % gene transfection efficiency at an MOI of 150. In addition, it significantly and specifically down-regulated TNF-alpha at both the mRNA and protein levels.

In addition to the dose, size, and shape of the particle, its material property is considered to be a major parameter influencing the consequent immune response [34]. With this background, the intervention discussed in this study was caused by two different wear particles, titanium and ceramic, which represent the most relevant and frequently observed biological sources of wear debris observed in clinical practice.

Under in vivo conditions, particles disperse randomly as they interact with the local tissue. The animal model used in our research was first described by Merkel, et al. [35]. Studies on small animals are cost-effective, quick, sensitive, and efficacious in screening agents with the potential to prevent the bone loss associated with wear particles. The murine calvarial osteolysis model serves as a simulation of the mechanisms responsible for the aseptic loosening observed in human joints. With desirable sensitivity and cost-effectiveness, its use in this study helps provide insight into the responses of inflammatory osteolysis induced by particles during aseptic loosening. However, there are documented limitations to the murine model [36]. In particular, the process of osteolysis in the murine calvarial model is by no means identical to that seen in human patients. Thus, the results of the study described here must be interpreted with caution, and future studies involving large animals and humans are necessary to confirm the efficacy of the agents tested in this model. Although the model established in our study only represented an acute setting, it nevertheless served as an efficient, sensitive, and cost-effective way to study the cellular and molecular responses of particle-induced inflammation. In other words, this model enabled us to efficiently demonstrate the expected results. As the optimal clinical protocol for wear particle induced inflammation has not yet been established, and any adverse effects of lentivirus treatment are yet to be thoroughly understood, further studies are warranted. In our murine cranial model, the particles were directly implanted in the periosteal space, instead of wearing off from a real prosthesis, as encountered in the ‘replace the joint with an implant’ scenario [37]. Due to the ready-to-use wear particles, the osteolysis was quickly evident. As for the delivery method, intravenous injection is the most widely used route to introduce lentivirus-mediated shRNA in vivo, because of its convenience and efficacy. However, the majority of lentivirus-mediated shRNA is not taken up by target tissues, making side effects inevitable due to the systemic down-regulation of the target gene. Recently, various methods of local siRNA administration have been suggested, which have been shown to result in higher silencing efficacy and fewer systemic side effects [38, 39]. The results from our study are consistent with this trend. All of these studies indicate that the local administration of lentivirus-mediated shRNA might be a promising strategy in the field of RNAi research. Studies in which lentiviral vectors are delivered directly to the murine calvaria are not yet available. In the present study, however, we came closer by introducing lentivirus-mediated shRNA via local injection, a modification made to the conventional administration method in the murine calvarial model.

By taking advantage of the strengths of murine calvaria, a lentiviral vector, and local administration, we explored the role of a novel gene therapy in inhibiting particle-induced inflammation and osteolysis. As shown by the experiments, a single dose of LV-mediated siRNA targeting TNF-alpha resulted in the hypoexpression of the corresponding mRNA by up to 80 %, and markedly compromised inflammatory responses in murine calvaria for at least 2 weeks. In addition, the fact that GFP fluorescence was found to be localized in the pouch area suggested that local administration was a safe and effective delivery route for the siRNA. Combined, these results lead to the conclusion that LV-mediated TNF-alpha shRNA remarkably inhibit particle-induced inflammation and osteolysis in the murine calvarial model. This finding serves as a building block for in vivo RNAi therapy in addressing aseptic loosening.

## Conclusions

In summary, our investigation shows that lentivirus-mediated shRNA targeting the TNF-alpha gene inhibits particle-induced inflammation and osteolysis both in vitro and in vivo. Conscious efforts have been made in this study to investigate an alternative therapeutic strategy for applying RNAi for the amelioration of this condition. Although the gap between this trial and clinical care for aseptic loosening has not yet been filled, this study has yielded insight into the potential therapeutic role of siRNA in this disease. In order to assess the osteolytic mediators in particle-induced aseptic loosening, the quantitative assessment of inflammatory cytokines on the murine calvarial surface is worthy of research.

## Abbreviations

CT: Threshold cycle
FBS: Fetal bovine serum
M-CSF: Macrophage colony stimulating factor
MOI: Multiplicities of infection
MPR: Multi-planar reformation
RANKL: Receptor activator nuclear factor κB ligand
SD: Standard deviation
shRNA: short hairpin RNA
siRNA: small interfering RNA
VR: Volume rendering
